# The effectiveness and safety of Sun tip-flexible ureterorenoscope for the management of kidney stones: A protocol for systematic review and meta-analysis of randomized controlled trials

**DOI:** 10.1097/MD.0000000000023964

**Published:** 2021-01-08

**Authors:** Yuecai Yuan, Rui Zhong, Haibiao Lai, Zhifeng Huang, Ye Zeng, Song Wu, Liang Zhong

**Affiliations:** a Zhongshan Hospital Affiliated to Guangzhou TCM University, Zhongshan, Guangdong Province; b The First Affiliated Hospital of China Medical University, Shenyang, Liaoning Province, P.R. China.

**Keywords:** kidney stones, meta-analysis, protocol, Sun tip-flexible ureterorenoscope, systematic review

## Abstract

**Introduction:**

Kidney stone is one of the urinary system diseases with a high incidence. In this study, we will evaluate the effectiveness and safety of Sun tip-flexible ureterorenoscope treating patients with kidney stone.

**Methods and analysis:**

English and Chinese literature about Sun tip-flexible ureterorenoscope treatment for kidney stones published before October 31, 2020 will be systematic searched in PubMed, Embase, Web of Science, Cochrane Library, Open Grey, Clinicaltrials.gov, Chinese Clinical Trial Registry, WANFANG, VIP Chinese Science and Technology Journal Database, CNKI, Chinese biomedical document service system (SinoMed). Only randomized controlled trials (RCTs) of patients with kidney stones will be included. Literature screening, data extraction, and the assessment of risk of bias will be independently conducted by 2 reviewers, and the 3rd reviewer will be consulted if any different opinions existed. Systematic review and meta-analysis will be produced by RevMan 5.3 and Stata 14.0. This protocol reported in accordance with the Preferred Reporting Items for Systematic Review and Meta-analysis Protocols (PRISMA-P) statement, and we will report the systematic review by following the PRISMA statement.

**Results:**

The current study is a protocol for systematic review and meta-analysis without results, and data analysis will be carried out after the protocol. We will share our findings in the fourth quarter of 2021.

**Conclusion:**

This study will provide recommendations for the effectiveness and safety of Sun tip-flexible ureterorenoscope for patients with kidney stones (KS), which may help to guide clinician.

**Ethics and dissemination:**

Ethical approval is not required as the review is a secondary study based on published literature. The results of the study will be published in peer-reviewed publications and disseminated electronically or in print.

**Protocol registration number:**

INPLASY2020110099

## 1 Introduction

Kidney stones (KS), also known as nephrolithiasis, is caused by abnormal accumulation of crystalline substances (such as calcium, oxalic acid, uric acid, cystine, etc) in the kidneys.^[1]^ It may be localized to any part of the urothelial system, causing common systemic symptoms, some of which may become acute. 40% to 75% of patients with kidney stone have different degrees of low back pain.^[2,3]^ The stones are large and the mobility is small, manifested as discomfort of soreness in the waist, or dull pain when physical activity increases.^[4]^ Colic caused by small stones, sudden abdomen-like pain in the abdomen and abdomen often occurs suddenly, showing paroxysmal. Urinary stone disease accounts for a considerable portion of the clinical workload for many urologists in this country and it consumes significant resources.^[5]^ With the development of social economy and changes in dietary habits, the incidence of kidney stone has become higher and higher in recent years.^[6,7]^ It has been estimated that its prevalence rates are up to 14.8% and increasing, and its recurrence rates are up to 50% within the subsequent 5 to 10 years after the first episode.^[8,9]^ If it cannot be treated effectively, it can cause significant morbidity, and can seriously impact quality of life in patients with kidney stones.^[10–12]^

A variety of managements for kidney stones are available, such as acupuncture, herbal medicine, surgery, dietary supplementation, oral medicine, and extracorporeal shock wave lithotripsy (ESWL).^[13–20]^ Surgical methods include percutaneous nephrolithotomy (PCNL) and retrograde intrarenal surgery (RIRS). Sun tip-flexible ureterorenoscope belongs to flexible ureteroscope, and is a novel combined ureteroscope created and invented by Chinese doctor Yinghao Sun and his team. The innovative combination of a hard scope with a flexible end and a retractable outer sheath enables the flexible ureteroscope to bend bidirectionally and turn coaxially. Randomized controlled trial (RCTs) provide the most reliable evidence for medical intervention,^[21]^ and the quality of evidence is higher than that of observational studies.^[22]^ Several RCTs have been conducted, evaluating the effects of Sun tip-flexible ureterorenoscope. However, the majority these trials have been single center. Therefore, we need to conduct a systematic review and meta-analysis of RCTs to evaluate the safety and efficacy of Sun tip-flexible ureterorenoscope for patients with kidney stones.

## 2 Materials and methods

This protocol refers to the statement of Preferred Reporting Items for Systematic Review and Meta-analysis Protocols (PRISMA-P).^[23,24]^ And we will report the systematic review by following the PRISMA statement. This protocol has been registered with the International Platform of Registered Systematic Review and Meta-analysis Protocols (registration number: INPLASY2020110099) which could be available on https://inplasy.com/.

### 2.1 Eligibility criteria

We will include studies according to the criteria outlined below.

#### 2.1.1 Type of studies

This study will include only RCTs. Other studies such as observational studies, retrospective analyses, self-controlled trials, patient series, case reports, reviews, animal studies, and laboratory in vitro studies will be excluded.

#### 2.1.2 Type of participants

All included participants must be diagnosed with KS, regardless of country, ethnic background, sex, age, and economic status.

#### 2.1.3 Type of interventions

Any forms of Sun tip-flexible ureterorenoscope intervention alone have been assigned to the patients in the experimental group. The intervention in the control group could be any management, except Sun tip-flexible ureterorenoscope.

#### 2.1.4 Type of outcomes

The primary outcomes is overall stone-free rate. The secondary outcomes are mean stone size (mm), pain intensity, urinary biochemical variables, mean hospital stay (day), quality of life, and adverse events.

### 2.2 Search methods

#### 2.2.1 Information sources

PubMed, Science Citation Index, Embase (Ovid) database, the Cochrane Library, and 4 Chinese databases (the China National Knowledge Infrastructure, the China Biology Medicine disc, the China Science and Technology Journal Database, and the Wan fang Database) will be searched from database inception to October 31, 2020. ClinicalTrials.gov and the Chinese Clinical Trial Registry Platform will be searched for ongoing or recently completed trials. Besides, we will scan the reference lists of included studies or relevant reviews to identify additional eligible studies, while the papers and unpublished reports will be hand-searched to ensure more complete coverage of the topic.

#### 2.2.2 Search strategies

Subject heading, lower words, entry terms, and free words search will be used in PubMed, Embase, and Cochrane library. Cochrane library search will be restricted by using “search word variations.” Topic search will be used in Web of Science. Free words will be searched within title, abstract, keywords in Cochrane library, Embase and within title, and abstract in PubMed. Chinese database search: CNKI will be restricted by using “topic” field; WANFANG and VIP will be limited by “title or keyword” filed; SinoMed will be searched by using subject words search plus synonym retrieval.

Search terms include: “Kidney Calculi” or “Kidney Stones” or “Nephrolith” or “Nephrolith” and “Sun's tip-flexible ureterorenoscope” or “sun's mirror” or “Sun's ureteroscope” or “Sun's Tip-Flexible Rigid Ureterorenoscopy.” Chinese search will use the Chinese form of the above terms. The example of specific search for PubMed is shown in Table 1.

**Table 1:** Example of PubMed search strategy.

Number	Search terms
#1	Sun's tip-flexible ureterorenoscope[Mesh] OR sun mirror[All Fields] OR Sun's ureteroscope [All Fields] OR a rigid ureteroscope with a deflectable tip[All Fields] OR Sun's Tip-Flexible Rigid Ureterorenoscopy[All Fields]
#2	Kidney Calculi[Mesh] OR Calculi, Kidney[All Fields] OR Calculus, Kidney[All Fields] OR Kidney Calculus[All Fields] OR Nephrolith[All Fields] OR Renal Calculus[All Fields] OR Kidney Stones[All Fields] OR Kidney Stone[All Fields] OR Stone, Kidney[All Fields] OR Stones, Kidney[All Fields] OR Renal Calculi[All Fields] OR Calculi, Renal[All Fields] OR Calculus, Renal[Title/Abstract]
#3	#1 AND #2

### 2.3 Data collection

#### 2.3.1 Selection of studies

**Figure 1:**
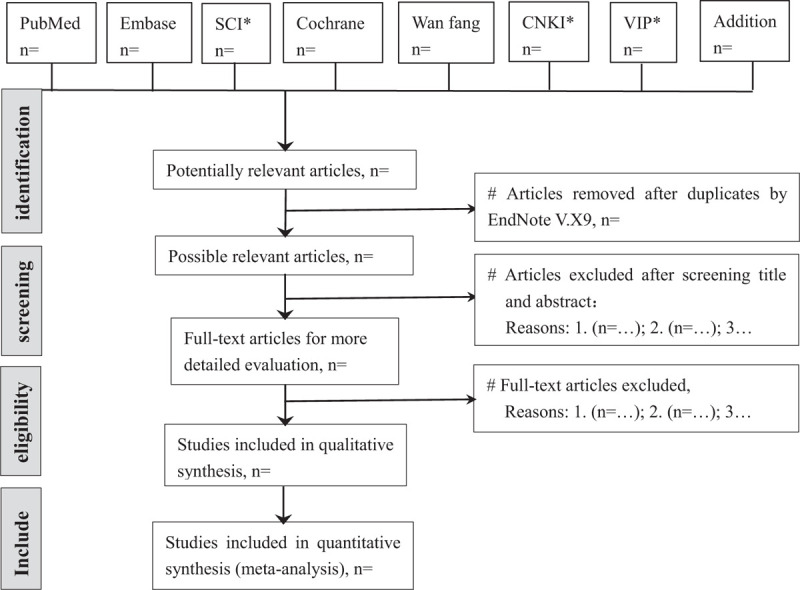
Study selection flow chart. CBM = China Biology Medicine disc; CNKI = China National Knowledge Infrastructure; SCI = Science Citation Index; VIP = China Science and Technology Journal Database.

According to pre-defined eligibility criteria, the screening will be carried out in duplicate by 2 independent reviewers (YY and RZ) at each stage of the review. Studies will be removed if they don’t meet the inclusion criteria obviously. If the studies appear to meet the inclusion criteria or there is any uncertainty based on the information provided in the title and abstract, full texts will be obtained for further assessment. When necessary, we will contact the author for more details of the study to solve questions about eligibility. Disagreements will be resolved by discussion or consulting expert (LZ) for arbitration. The number and reasons for excluding trials will be recorded in detail. A flow diagram of the study selection is shown in Fig. 1.

#### 2.3.2 Data extraction

Data extraction for eligible studies will be performed independently by 2 reviewers (YY and RZ) using a pre-designed standardized form. We will provide guidance and interpretation for the contents of the extraction form before data extraction. The detailed data extraction form will mainly consist of basic information, population characteristics, methodological description, intervention characteristics, outcome data, conclusion, and follow-up assessment. We will contact the original researchers for missing data. The third reviewer (ZH) will be responsible for checking the data extracted by the 2 reviewers. Inconsistencies will be resolved by discussion, and consulting the superior expert (LZ) to facilitate the decision when a disagreement persisting.

#### 2.3.3 Assessment of risk of bias

The methodological quality of individual studies will be judged following the criteria from the Cochrane Handbook for Systematic Reviews of Interventions Version 5.3.0.^[25]^ The judgments of all included studies will be made independently by 2 reviewers (YY and RZ), and we will conduct training of reviewers and calibration exercises before the start of the review to ensure consistency between reviewers. There are 7 domains, each of which will be rated as “yes” (indicating a low risk of bias), “no” (indicating a high risk of bias), or “unclear” (indicating either an uncertainty for bias or lack of information). The original study investigators will be contacted if any uncertainty exists. We plan to compute graphic representations of potential bias within and across studies using Review Manager 5.3. Those with inconsistent opinions will be resolved through negotiation or consult the superior expert (LZ) to reach a consensus. Overall, the following aspects will be considered:

(1) Appropriate generation of random allocation sequence (selection bias);
(2) Concealment of the allocation sequence (selection bias);
(3) Blinding of participants and healthcare providers (performance bias);
(4) Blinding of data collectors and outcome adjudicators (detection bias);
(5) Incomplete outcome data such as dropouts and withdrawals (attrition bias);
(6) Selective outcome reporting (publication or dissemination bias);
(7) Other bias (such as sponsorship bias).

### 2.4 Data analysis

#### 2.4.1 Data synthesis and meta-analysis

We will perform a systematic narrative synthesis to summarize and explain the characteristics and findings of the included studies and provide this information in the text and tables. Review Manager 5.3 provided by the Cochrane Collaboration will be used for the meta-analysis (if feasible), and the random-effects model will be chosen to combine all summary outcome measures. If a meta-analysis is impossible, the results of clinical trial comparisons will be analyzed descriptively. Dichotomous outcomes (e.g., effective and ineffective) will be determined by relative risk (RR) with 95% confidence interval (CI), while continuous data will be analyzed using weighted mean difference (if measurement methods are consistent) or standardized mean difference (if measurement methods are different).

#### 2.4.2 Dealing with missing data

When there are missing data, we will contact the study authors via email to obtain detailed accurate data. If the missing data are not available finally, we will carefully estimate the important numerical data, for example using an interpolation method. Moreover, the potential impact of missing data on the overall results of the study will be assessed using sensitivity analysis. It is possible to include multiarm trials, we will combine the relevant groups into a single group according to the formula provided in the Cochrane handbook 5.3.0.^[25]^

#### 2.4.3 Assessment of heterogeneity and publication bias

Heterogeneity of each outcome measure will be tested using the Chi^2^ test and *I*^*2*^ statistic.^[26]^ If there is significant heterogeneity among the trials (*I*^2^ ≥ 50% or *P* < .1), we will try to explain the source of heterogeneity through subgroup analysis or sensitivity analysis. And we should not perform a meta-analysis if heterogeneity is substantial, a narrative qualitative summary will be done instead. Funnel plot will be used to reveal potential publication bias if over 10 studies are available.^[27]^

#### 2.4.4 Subgroup analysis and sensitivity analysis

When there is obvious heterogeneity among included studies, we will perform a subgroup analysis in accordance with different study qualities, treatments, controls, and outcome measurements. We will also use sensitivity analysis to test the stability and reliability of meta-analysis. It will be conducted by 2 methods: eliminating each study one by one; using random-effect model (DerSimonian and Laird method) to test the results after using the fixed effect model.^[28,29]^

### 2.5 Grading the quality of evidence

The quality of evidence in the systematic review will be judged by the GRADE tool.^[30]^ It is based on 5 key domains: risk of bias, consistency, directness, precision, and publication bias. The evidence levels for each outcome will be adjudicated as high quality, moderate quality, low quality, and very low quality.^[31]^ RCTs with low risk of bias are considered high-quality evidence that could provide a direct and precise reference for clinical application.

### 2.6 Reporting of the review

The methodological quality of the systematic review and meta-analysis to be completed next will be standardized by each item of the AMSTAR-2 tool.^[32]^ And the results will be reported following the Preferred Reporting Items for Systematic Reviews and Meta-Analysis (PRISMA) statement published in 2009.^[33]^

## 3 Discussion

The prevalence of urinary lithiasis varies between 1% and 20% worldwide.^[34]^ In recent years, the incidence has increased due to several factors: geographical, climatic, nutritional, and genetic. Currently, it is one of the primary urological diseases necessitating treatment.^[35]^ Flexible and semirigid ureteroscopy (URS) techniques have become the standard of care in the management of ureteral and renal stones, supported by urologic guidelines.^[36,37]^ Sun tip-flexible ureterorenoscope belongs to flexible ureteroscope, and is a novel combined ureteroscope created and invented by Chinese doctor Yinghao Sun and his team. This systematic review and meta-analysis will evaluate the effectiveness and safety of Sun tip-flexible ureterorenoscope treating patients with KS. The results of this review will be widely disseminated through peer-reviewed publications and conference presentations. This evidence may also provide helpful evidence of whether Sun tip-flexible ureterorenoscope would have better curative effect for patients with KS.

This systematic review has the following limitations: First, as we are not good at other languages, the literatures we searched are limited to Chinese and English, which will cause certain bias. Second, there may be a limited number and sample size of RCT for treating kidney stones, the quality of evidence provided may not be high. Third, the limitation of sample size also leads to the instability of conclusion reliability. Therefore, we hope that there will be more large-scale, multicenter, high-quality RCTs in the future to provide high-quality evidence.

## Author contributions

**Conceptualization:** Yuecai Yuan.

**Data curation:** Yuecai Yuan, Rui Zhong, Zhifeng Huang.

**Formal analysis:** Rui Zhong, Zhifeng Huang, Ye Zeng.

**Methodology:** Yuecai Yuan, Rui Zhong.

**Project administration:** Yuecai Yuan, Liang Zhong.

**Software:** Rui Zhong, Zhifeng Huang, Ye Zeng, Song Wu.

**Supervision:** Liang Zhong

**Validation:** Haibiao Lai, Zhifeng Huang, Ye Zeng.

**Writing – original draft:** Yuecai Yuan, Rui Zhong, Haibiao Lai.

**Writing – review & editing:** Yuecai Yuan, Rui Zhong, Liang Zhong.
